# Low Expression Levels of SLC22A12 Indicates a Poor Prognosis and Progresses Clear Cell Renal Cell Carcinoma

**DOI:** 10.3389/fonc.2021.659208

**Published:** 2021-06-23

**Authors:** Jiaju Xu, Yuenan Liu, Jingchong Liu, Yi Shou, Zhiyong Xiong, Hairong Xiong, Tianbo Xu, Qi Wang, Di Liu, Huageng Liang, Hongmei Yang, Xiong Yang, Xiaoping Zhang

**Affiliations:** ^1^ Department of Urology, Union Hospital, Tongji Medical College, Huazhong University of Science and Technology, Wuhan, China; ^2^ Department of Pathogenic Biology, School of Basic Medicine, Huazhong University of Science and Technology, Wuhan, China; ^3^ Shenzhen Huazhong University of Science and Technology Research Institute, Shenzhen, China

**Keywords:** cellular homeostasis, renal cell carcinoma, biomarker, gene set enrichment analysis, metastasis, solute carrier family, bioinformatic analysis, signal pathway

## Abstract

Clear cell renal cell carcinoma (ccRCC) accounts for approximately 4/5 of all kidney cancers. Accumulation of minor changes in the cellular homeostasis may be one cause of ccRCC. Therefore, we downloaded the RNA sequencing and survival data of the kidney renal cell carcinoma (KIRC) cohort from the Cancer Genome Atlas (TCGA) database. After the univariate and multivariate Cox regression analyses, 19 kidney-specific differentially expressed genes (DEGs) were found. Solute Carrier Family 22 Member 12 (SLC22A12) resulted in an independent prognostic predictor for both overall survival (OS) and disease-free survival (DFS). SLC22A12 expression was lower in tumoral tissue compared to normal tissue. Moreover, patients in the SLC22A12 low expression group had a higher pathological stage and worse survival than the high expression group. Additionally, qRT-PCR assay, immunoblotting test (IBT), and immunohistochemical (IHC) analyses of cancer tissues/cells and the corresponding normal controls verified that SLC22A12 is downregulated in ccRCC. Receiver operator characteristic (ROC) curves showed that the low expression level of SLC22A12 could be a good diagnostic marker for ccRCC (AUC=0.7258; p <0.0001). Gene set enrichment analysis (GSEA) showed that SLC22A12 expression levels are related to metabolism, cell cycle, and tumor-related signaling pathways. GO and KEGG analyses revealed that SLC22A12 transports multiple organic compounds, ions, and hormones and participates in the extracellular structure organization. Furthermore, SLC22A12 over-expression *in vitro* inhibited the proliferation, migration, and invasion of renal cancer cells by regulating PI3K/Akt pathways. Such effects were reversed when knocking out SLC22A12. In summary, as a transporter for many vital metabolites, SLC22A12 may affect tumor cell survival through its impacts on the mentioned metabolites. In conclusion, this study uncovered that SLC22A12 is a promising prognostic and diagnostic biomarker for ccRCC.

## Introduction

Kidney cancer, also called renal cancer, represents a significant threat to human health, as it develops fatal metastasis in the lung or brain. Kidney cancer mainly includes renal cell cancer (RCC), transitional cell cancer (TCC), and Wilms tumor. RCC accounts for roughly 4/5 of kidney cancers, while most of the other renal cancer are TCC (1–3). According to the American Cancer Society, 73,750 new kidney and renal pelvis cancer cases were estimated in the United States in 2020. Among the diagnosed, 520 were males and 28,230 females, with an estimated 14,830 deaths, including 9,860 males and 4,970 females (4). The five-year survival rate is 93% for patients with localized kidney cancer, 70% with surrounding lymph nodes spread, and 12% with distant metastasis (5). Surgery is the typical treatment for kidney cancer due to the slight response to radiation and chemotherapy. Target therapy is growing, but its capacity to limit the progression is restricted. Therefore, it is crucial to investigate the biological functions and molecular mechanisms involved in kidney cancer in order to find new therapeutic targets.

Maintaining homeostasis is essential for a healthy body, and the same happens in the constituent cells. Complex intracellular reactions occur to maintain the level of various biological macromolecules, including multiple enzymes, transporters, kinases, and cytokines. The environment where epithelial cells live is more complicated than normal cells. Since there is an active exchange of substances, a considerable accumulation of metabolic waste, and a distinctive osmotic pressure difference, it is more challenging to maintain cellular homeostasis. During the adaptation to this harsh environment, epithelial cells have developed a cloning strategy to replace the exfoliated cells. This strategy contributes to dyshomeostasis, tumorigenesis, and differential protein expression profile. Unfortunately, the connection between cellular homeostasis and tumorigenesis has not been thoroughly studied. Since we consider this a critical pathophysiological aspect, we aim to study oncogenes or tumor suppressor genes specifically expressed in the kidney. Finding differentially expressed genes in kidney cancer will contribute to a deeper understanding of this cancer and could also help to identify new prognostic biomarkers. Moreover, potential new drugs against these targets may be safer since they will not harm other organs and tissues.

We found that Solute Carrier Family 22 Member 12 (SLC22A12), a tissue-specific gene, played an important role in the occurrence and development of kidney cancer. SLC22A12, also known as Urate Transporter 1(URAT1) or Renal-Specific Transporter (RST), is a membrane protein located in epithelial cells of the kidney proximal tubule. Initially, it was considered a urate transporter (6–10); however, later studies proved that it is involved in pharmacodynamics (11–14). It can transport glucose and other sugars, bile salts and organic acids, metal ions and amine compounds (10, 13, 15–18). To the best of our knowledge, the relationship between SLC22A12 and ccRCC has not been disclosed yet.

We screened the TCGA data set to discover differentially expressed genes (DEGs) in kidney cancer. As a result, we found 19 kidney-specific genes that regulate cell homeostasis. Using Cox regression analyses, we discovered that SLC22A12 has a significant impact on patient survival. Our results demonstrate that SLC22A12 low expression predicted a poor prognosis in ccRCC. Additionally, an *in vitro* experiment confirmed its role as a tumor suppressor. Gene set enrichment analysis, and protein-protein interaction (PPI) network strengthened the hypothesis that SLC22A12 contributes to the homeostasis regulation in ccRCC.

## Materials and Methods

### Dataset

The data were gathered from the Cancer Genome Atlas (TCGA) project (https://portal.gdc.cancer.gov/), cBioPortal for Cancer Genomics (http://www.cbioportal.org/), UCSC Xena browser (https://xenabrowser.net/), and International Cancer Genome Consortium (ICGC, https://dcc.icgc.org/), including gene expression datasets (RNA sequencing, RNA‐seq) on kidney renal clear cell carcinoma (KIRC) patients, as well as corresponding demographic (age, gender), clinicopathological [American Joint Committee on Cancer (AJCC) T stage, N stage, M stage, G stage and clinical stage] and survival (overall survival, disease-free survival) information (19, 20). Patients without survival information were eliminated from further evaluation.

### Screening of the Critical Gene Involved in Cellular Homeostasis in ccRCC

The gene set of cellular homeostasis was downloaded from the Gene Ontology (GO) Resource (http://geneontology.org/). Kidney-specific gene set was gathered from the Human Protein Atlas (https://www.proteinatlas.org/). Differential expressed gene set was acquired by “limma” package (21–23) with a cut-off value of p<0.05 by R 4.0.2. Then a Venn diagram was depicted to obtain the intersection of three gene sets for further study. Pan-cancer profile of SLC22A12 was gathered from Gene Expression Profiling Interactive Analysis (GEPIA, http://gepia.cancer-pku.cn/) (24).

### ccRCC Tissue Samples

A total of 120 pairs of ccRCC and their adjacent normal renal tissues were sampled from patients aged 22-79 years old between May 2015 and May 2018 at the Department of Urology, Union Hospital, Tongji Medical College, Huazhong University of Science and Technology (Wuhan, China). The adjacent normal renal tissues were collected more than 2 cm away from the edge of the tumor site. The proteins extracted from 16 pairs of these resected samples were analyzed *via* immunoblotting test. The RNAs extracted from 20 pairs of samples were analyzed *via* reverse transcription-quantitative PCR (qRT-PCR). Two pairs of tissues were analyzed *via* immunohistochemistry (IHC). Three pairs of tissues were analyzed by whole transcriptome sequencing in 2017. The basic clinical characteristics (age, gender, tumor size, tumor location and tumor stage) of the patients are presented in **Table S2** (25). No patients had received any adjuvant anticancer therapy prior to or following surgery. The present study was approved by the Human Research Ethics Committee of Huazhong University of Science and Technology. Written informed consent was provided by the patients or the patients’ family. The study methodologies conformed to the standards set by the Declaration of Helsinki.

### Cell Culture

The human renal proximal tubular epithelial cell line HK-2, and five types of human renal cell carcinoma cell lines purchased from the American Type Culture Collection (ATCC, USA), including 786-O, ACHN, A-498, OSRC-2 and Caki-1, were employed in the present study. The cells were cultured in high glucose Dulbecco’s Modified Eagle’s Medium (DMEM; Gibco, USA) containing 10% fetal bovine serum (FBS; Gibco, USA) and 1% penicillin-streptomycin solution (Servicebio, China) and incubated in a humidified atmosphere with 5% CO_2_ at 37°C.

### Immunoblotting Test (IBT)

Cells and tissues were lysed in Radio Immunoprecipitation Assay (RIPA) Lysis Buffer (Beyotime, China) containing protease inhibitors. Then the protein concentration of each sample was measured using a BCA Protein Assay Kit (Beyotime, China). For IBT, 15 µg proteins were separated *via* SDS-PAGE (12% gel) at 90-120 mV for 90 min and transferred to a polyvinylidene difluoride (PVDF) membrane (Invitrogen, USA) at 300 mA for 60 min. Afterwards, the PVDF membranes were blocked with 2.5% bovine serum albumin (BSA) for 2 h at room temperature and then incubated with specific primary antibodies overnight at 4°C. The primary antibody used in this paper: anti-SLC22A12(14937-1-AP), 1:1,000, Proteintech, China; anti-GAPDH(AC002), 1:5,000, Abclonal, China; anti-PI3K(PAB43806), 1:2,000, Bioswamp, China; anti-p-PI3K(PAB43641-P), 1:2,000, Bioswamp, China; anti-AKT1(A17909), 1:3,000, Proteintech, China; anti-p-AKT1(ab81283), 1:3,000, Abcam, US. Following incubation with the primary antibodies, the membranes were incubated with specie-matched secondary antibodies (AS014/AS003, 1:3,000; Abclonal, China) for 2 h at room temperature following washing with PBST for 30 min. Finally, the protein bands were visualized with Electrochemiluminescence (ECL) Western Blotting Substrate (Ultra sensitivity; Biosharp, China) using ChemiDoc-XRS+ (Bio-Rad, China).

### RNA Extraction and qRT-PCR

Total RNA was isolated from tissues or cells using Ultrapure RNA Kit (CoWin Biosciences, China) directed by the manufacturer’s protocols. The concentration and purity of the RNA solution were detected using Tecan’s Infinite M200 Pro (Thermo Fisher Scientific, USA). Extracted RNA was then reverse transcribed into cDNA using PrimeScript™ RT Master Mix (Takara, Japan) according to the manufacturer’s protocols. The reaction conditions were as follows: 37°C for 15 min; 85°C for 5 sec. Subsequently, the cDNA was diluted at a proper concentration and subjected to qPCR using AceQ^®^ qPCR SYBR Green Master Mix (Vazyme, China) on CFX Connect Real-Time PCR Detection System (Biorad, China) according to the manufacturer’s protocols. The qPCR conditions were as follows: pre-denaturation at 95°C for 5 min; 40 cycles of denaturation at 95°C for 10 sec; annealing and extension at 60°C for 30 sec. The housekeeping gene, GAPDH, was used to normalize the relative expression of SLC22A12 as an endogenous control by the comparative Ct (threshold cycle) method (2^−ΔΔCt^). All qRT-PCR reactions were performed in duplicate. The primers used to amplify SLC22A12 and GAPDH were chemically synthesized by TSINGKE, China. The primer sequences were as follows: SLC22A12: 5′- TCT CCA CGT TGT GCT GGT TC -3′ (forward) and 5′- GGA TGT CCA CGA CAC CAA TGA -3′(reverse); GAPDH: 5′- CGT GGA AGG ACT CAT GAC CA -3′ (forward) and 5′- GCC ATC ACG CCA CAG TTT C -3′ (reverse).

### Immunohistochemistry (IHC) Assay

The IHC assay was performed as previously described. Briefly, ccRCC tissues and adjacent normal tissues were sequentially fixed in formalin at room temperature for 12 h, dehydrated and embedded in paraffin. Tissue sections were then incubated with a rabbit antibody against SLC22A12 overnight at 4°C. They were then rinsed three times with PBS and incubated with secondary antibodies that were conjugated to horseradish peroxidase at room temperature for 2 h. Finally, tissues were observed in three randomly selected fields under a light microscope (Olympus CX41-32C02; Olympus, Japan) at 40, 100, and 200× magnification.

### Transient Transfection for Overexpression and Knockdown of SLC22A12

Plasmids overexpressing SLC22A12 and a negative control (Vector) were constructed by Vigene Biosciences (Shandong, China). Small interfering RNA (siRNA) oligonucleotide sequences specifically targeting SLC22A12 (si-SLC22A12) and a negative control (si-NC) siRNA were synthesized by Guangzhou RiboBio and verified no off-target effects by Basic Local Alignment Search Tool (BLAST, https://blast.ncbi.nlm.nih.gov/Blast.cgi). For transient transfection, ACHN and 786-O cell lines were incubated in 6-well plates until they reached 70% confluence. 10μg per well of plasmids (vector or SLC22A12) or 0.1 nmol per well of siRNAs (si-SLC22A12 or si-NC) were transfected with Invitrogen Lipofectamine^®^ 2000 (Thermo Fisher Scientific, USA) according to the manufacturer’s protocol. Cells were collected for subsequent experiments 48 h post-transfection. The si-SLC22A12 sequence was as follows: 5′- TCA CCT GCA TCA CCA TCT A -3′.

### Colony Formation Assay

ACHN and 786-O cells had been transfected with plasmids or siRNAs for 48 h before subsequent experimentation. Cells were inoculated on 6-well plates at a cell density of 1×10^3^ cells per well with 2 mL of medium. After culture for 10 days, cells were fixed with methanol for 10 min and stained with crystal violet for 20 min. After PBS wash and air drying, colonies (>50 cells) were manually counted. All experiments were independently repeated in duplicate.

### 5-Ethynyl-2′-deoxyuridine (EdU) Assay

EdU assay was implemented in ACHN and 786-O cells according to manufacturer’s protocol by use of the BeyoClick™ EdU-647 Cell Proliferation Kit (Beyotime, China). After transfection, cells (1 × 10^5^ per well) were seeded into 6-well plates. 24 h later, 10μM EdU medium was added into cells for 2 h. Next, 4% paraformaldehyde (PFA) was added for 15-min fixing. After three-time rinse by washing buffer (3% BSA), cells were washed by permeabilization buffer (0.3% Triton X-100) for 15 min. After one-time rinse by washing buffer, Click additive reaction system was added to label the proliferated cells and Hoechst 33342 was added for cell counting. Finally, cells were visualized using fluorescence microscope (Olympus, Japan). Each independent experiment was carried out in duplicate.

### Cell Counting Kit-8 (CCK8) Assay

ACHN and 786-O cells had been transfected with plasmids or siRNAs for 48 h before subsequent experimentation. Cells were inoculated on 96-well plates at a cell density of 1×10^3^ cells per well with 100 µl of medium. A cell proliferation assay was performed using Cell Counting Kit-8 (CCK8; MedChemExpress, USA) at a concentration of 10μl in 100μl serum-free medium every 24 h for four days according to the manufacturer’s protocols. After incubation for 2 h at 37°C, the optical density of each well was measured at 450 nm with a spectrophotometer to measure the quantity of living cells. Finally, the absorbance of cells over four days were plotted in a graph for a reflection of cell proliferation rate.

### Cell Migration and Invasion Assays

ACHN and 786-O cells had been transfected with plasmids or siRNAs for 48 h before subsequent experimentation. Prior to the assays, cells were incubated in serum-free DMEM for 6-8 h. Boyden Transwell chambers and 24-well plates (Corning, USA) with 8-µm membrane filters were used in the migration and invasion assays. Serum-starved cells (1×10^5^) were seeded into the upper chambers in serum-free medium, and the lower chambers were filled with DMEM containing 10% FBS. After incubation for 24 h at 37°C, the lower chamber was washed twice with PBS and fixed with 100% methanol for 10 min at room temperature and stained with 0.1% crystal violet dye for 20 min at room temperature. Following washing the chamber again three times with PBS, non-migrated and non-invaded cells were carefully removed from the upper chamber with a cotton bud. Migrated cells in lower chambers were observed in five randomly selected fields under a light microscope (Olympus CX41-32C02; Olympus, Japan) at 400× magnification. Based on the migration assay, a cell invasion assay was performed in Matrigel-coated Transwell insert chambers (BD Biosciences, USA), which had already been incubated at 37°C for 6-8 h, with double cell numbers. The remaining procedure was the same as described for the cell migration assays.

### Bioinformatics Analyses

The median of SLC22A12 expression was set as the cutoff point for dividing patients into high and low expression groups. To determine which SLC22A12 signaling pathways were involved in the pathogenesis of ccRCC, a gene set enrichment analysis (GSEA; http://www.broadinstitute.org/gsea) was used with the curated gene sets (c2.all.v7.1.symbols.gmt) that integrate Kyoto Encylopedia of Genes and Genomes (KEGG), Biocarta Pathways dataset, Reactome Pathway Database and Pathway Interaction Database (PID). For the enriched gene sets, after performing 1,000 permutations, the false discovery rate (FDR) value <0.25 and the p<0.05 were considered statistically significant enriched pathways (26). The KEGG and Gene Ontology (GO) analyses of DEGs between high and low SLC22A12 expression groups were conducted by R 4.0.2.

### Statistical Analyses

As seen in the previous article (27), statistical analyses were performed using GraphPad Prism version 7.0. The numerical data of each group are presented as the mean ± standard deviation. The significant differences in SLC22A12 expression between each ccRCC subgroup were analyzed using a Student’s t-test. A paired Student’s t-test was used to analyze SLC22A12 expression in tumor tissues and matched normal kidney tissues. The associations between SLC22A12 expression and clinicopathological characteristics in patients with ccRCC were evaluated using Pearson’s χ^2^ test. Receiver operator characteristic (ROC) curves and areas under the curve (AUC) were used to calculate the diagnostic values of SLC22A12 expression in patients with ccRCC. The association between SLC22A12 expression and OS was investigated using Kaplan-Meier curves with log-rank tests. p<0.05 was considered a statistically significant difference.

## Results

### SLC22A12 Is an Essential Kidney-Specific Tumor Suppressor Gene That Maintains Cell Homeostasis

**Figure 1:**
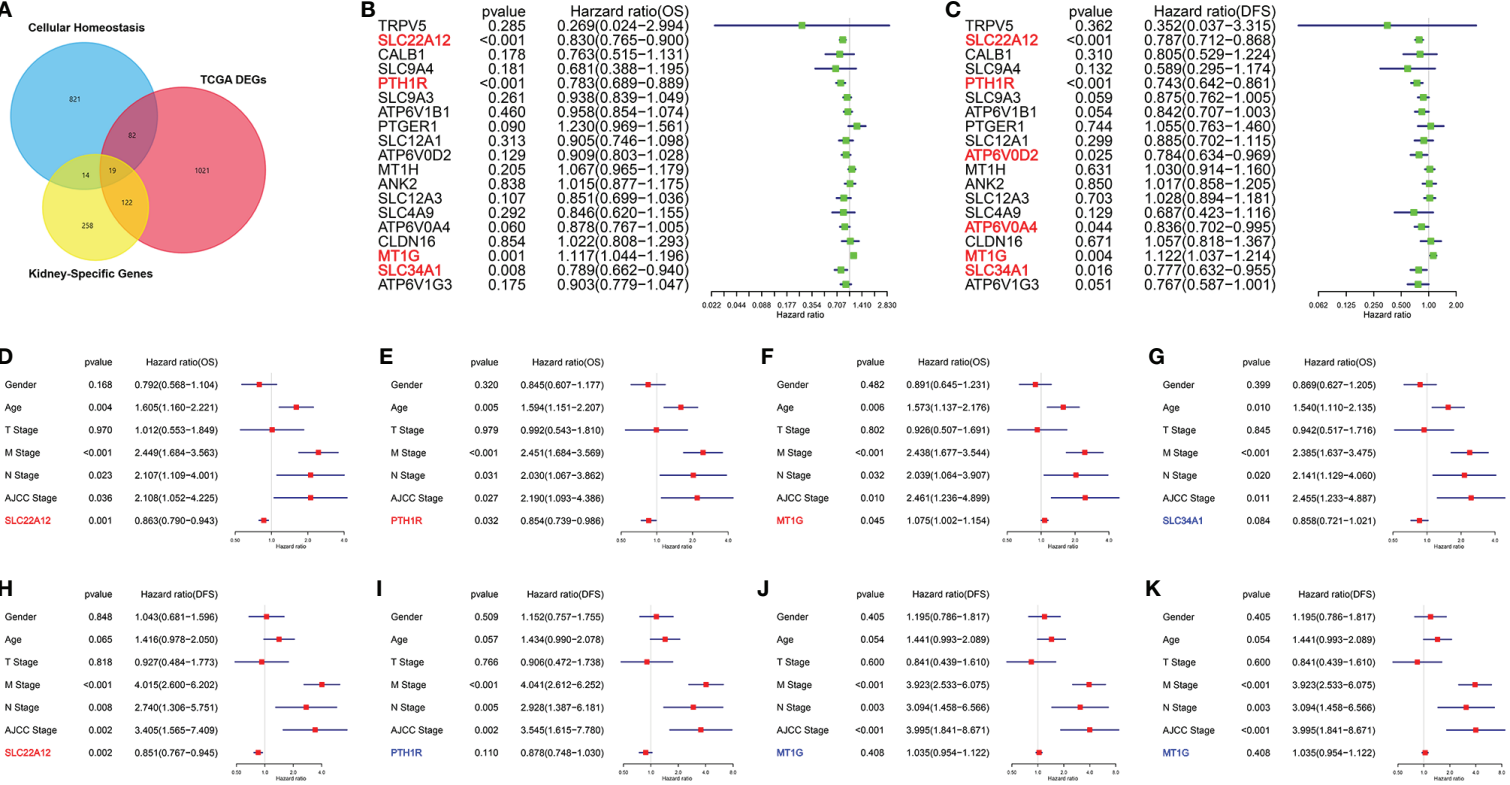
Screening of critical genes involved in cellular homeostasis in ccRCC. **(A)** Venn diagram selects 19 kidney-specific DEGs involved in cellular homeostasis. **(B, C)** Univariate Cox regression analyses of 19 selected genes select SLC22A12, PTH1R, MT1G and SLC34A1 as four prognostic biomarkers. Left: OS; right: DFS. **(D–K)** Multivariate Cox regression analyses of four candidate prognostic biomarkers along with clinicopathological characteristics select SLC22A12 as the critical gene. Middle row: OS; bottom row: DFS.

**Figure 2:**
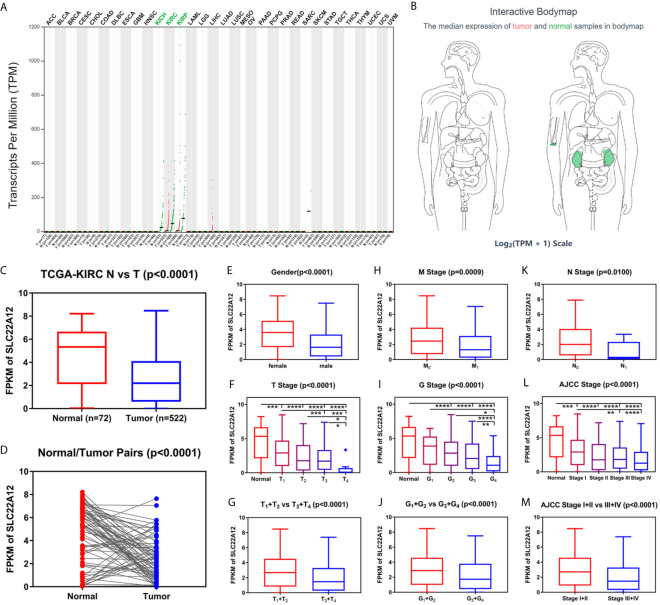
Transcriptome profile of SLC22A12 of kidney cancer in TCGA database. **(A)** Transcriptome profile of SLC22A12 in multiple types of cancers and their corresponding normal tissues in TCGA database. **(B)** Distribution of SLC22A12 RNA expression levels across organs. The mRNA expression levels of SLC22A12 were lower in **(C)** 522 ccRCC tissues than in 72 para-cancer tissues, **(D)** 72 ccRCC tissues than in 72 corresponding adjacent normal tissues. SLC22A12 expression was lower in **(E)** male, **(F, G)** higher T stage, **(H)** higher M stage, **(I, J)** higher G stage, **(K)** higher N stage, and **(L, M)** higher AJCC clinical stage. *p < 0.05, **p < 0.01, ***p < 0.001, ****p < 0.0001.

One of the aims of this work was to identify the critical genes for cellular homeostasis in ccRCC. For that purpose, we screened three independent gene sets: DEGs set in TCGA datasets, kidney-specific gene sets, and cellular homeostasis gene sets. As a result, we found that only 19 genes belonged to the three gene sets (**Figure 1A**). Next, univariate Cox regression analyses were performed to explore the relationship between the gene expression levels and patient survivals. As shown in **Figures 1B, C**, among all 19 genes, only SLC22A12, PTH1R, MT1G, and SLC34A1 expression significantly impacted overall survival (OS) and disease-free survival (DFS). Multivariate Cox analyses (**Figures 1D–K**) and clinicopathological data confirmed that SLC22A12 is an independent risk factor in both OS and DFS and has a better prognostic value. No other cancer transcriptome profile included SLC22A12, except for two sarcoma samples (SARC) (**Figures 2A, B**) (24). Apart from that, SLC22A12 was only expressed in the kidney (KIRC: Kidney renal clear cell carcinoma; KIRP: Kidney renal papillary cell carcinoma; KICH: Kidney Chromophobe). From the results above, SLC22A12 was selected as the principal gene for further investigation.

### SLC22A12 Downregulation Is Associated With Various Types of Clinicopathological Characteristics in ccRCC

Data of SLC22A12 mRNA expression levels in ccRCC tissues and para-cancer tissues were downloaded from the TCGA database to understand the role of SLC22A12 expression in tumorigenesis. The results suggest that SLC22A12 expression in tumor tissues was significantly lower than in para-cancer tissues (**Figures 2C, D**). Similar results were found in the ICGC database (**Figures S1A, B**). In addition, we studied the connection between SLC22A12 expression and the clinicopathological characteristics. We found that a decrease in the gene expression was associated with increasing primary tumor (T stage), regional lymph node (N stage), distant metastasis (M stage), histologic grade (G stage), and AJCC prognostic stage (**Figures 2F–M**). Also, SLC22A12 expression was lower in females (**Figure 2E**).

### SLC22A12 Downregulation Indicates a Poor Clinical Prognosis

**Figure 3:**
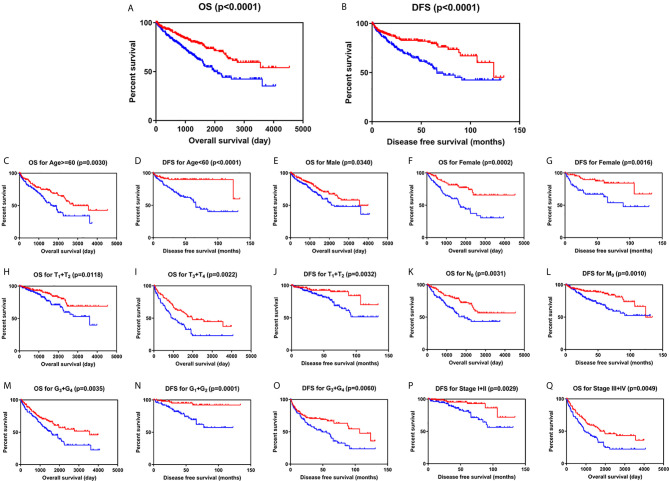
Low SLC22A12 mRNA expression is associated with both poor OS and DFS in patients with ccRCC. Patients with lower SLC22A12 mRNA expression levels harbor worse **(A)** OS and **(B)** DFS. The similar results were obtained from **(C–Q)** subgroup of patients with ccRCC.

Kaplan-Meier survival analysis with log-rank test was applied to determine the association between patients’ survival and SLC22A12 expression. From the TCGA database, 522 patients with ccRCC were divided into two groups using the SLC22A12 mRNA expression median as the cutoff criteria. The results revealed that the lower SLC22A12 expression group had the poorest OS and DFS (**Figures 3A, B**). The survival data from the ICGC-RECA cohort showed a similar pattern of results (**Figure S1C**). Kaplan-Meier survival analyses regarding SLC22A12 expression in ccRCC patients with different clinicopathological characteristics were in line with the previous results (**Figures 3C–Q**). The present findings indicate that SLC22A12 could have a prognostic value in ccRCC since its decreased expression resulted in poor patient outcomes.

### SLC22A12 Expression Levels Could Be Valuable for ccRCC Clinical Diagnosis

**Figure 4:**
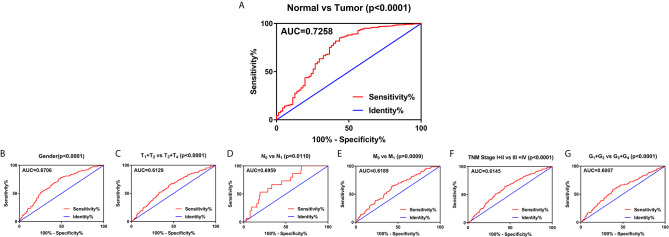
SLC22A12 expression may be a diagnostic predictor in patients with ccRCC. **(A)** SLC22A12 effectively discriminated between ccRCC and normal tissues. Receiver operating characteristic curve subanalysis were performed for the following subgroups of patients with ccRCC: **(B)** gender, **(C)** T stage, **(D)** N stage, **(E)** M stage, **(F)** AJCC clinical stage, **(G)** G stage. AUC, area under curve; OS, overall survival; DFS, disease-free survival.

To explore the diagnostic value of SLC22A12 in ccRCC, ROC curves were plotted to assess the clinicopathological characteristics of the patients. In general, ccRCC could be properly differentiated from normal tissues using SLC22A12 expression levels with an AUC of 0.7258 (p < 0.0001; **Figure 4A**) in the TCGA-KIRC cohort and 0.8926( p< 0.0001; **Figure S1D**) in the ICGC-RECA cohort. Furthermore, the diagnostic value of SLC22A12 expression levels was analyzed between clinicopathological subgroups: male vs. female (AUC=0.6706; p < 0.0001; **Figure 4B**); T_1_ + T_2_ vs. T_3_ + T_4_ stage (AUC=0.6129; p < 0.0001; **Figure 4C**); N_0_ vs. N_1_ stage (AUC=0.6959; p=0.0110; **Figure 4D**); M_0_ vs. M_1_ stage (AUC=0.6189; p=0.0009; **Figure 4E**); AJCC stage I + II vs. stage III + IV (AUC=0.6145; p < 0.0001; **Figure 4F**); G_1_+G_2_ vs. G_3_+G_4_ (AUC=0.0.6007; p < 0.0001; **Figure 4G**). Conclusively, SLC22A12 may be a potential diagnostic biomarker for clear cell renal cell carcinoma.

### SLC22A12 Is Down-Regulated in ccRCC Cells and Tissues

**Figure 5:**
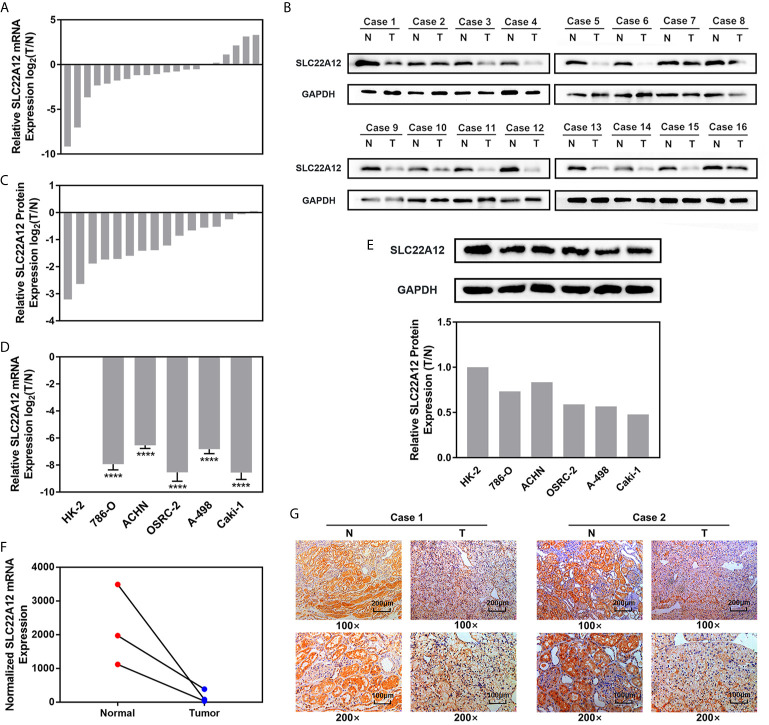
SLC22A12 was down-regulated in RCC cells and tissues compared to their corresponding control. Relative mRNA expression of SLC22A12 was lower in RCC **(A)** tissues and **(D)** cells than their normal control measured by qRT-PCR assays. Relative protein expression of SLC22A12 was lower in RCC **(B, C)** tissues and **(E)** cells than their normal control measured by immunoblotting tests. **(F)** Transcriptomic levels of SLC22A12 in normal and tumoral tissues gathered from Wuhan Union Hospital’s cohort. **(G)** Representative images of immunohistochemical analyses suggested a lower SLC22A12 expression in tumoral tissue. ****p < 0.0001.

qRT-PCR and IBT were performed to verify the expression levels of SLC22A12 in RCC cells. SLC22A12 mRNA and protein expression levels in RCC cell lines (786-O, ACHN, A-498, OSRC-2, and Caki-1) were decreased compared to the normal cell line HK-2 (**Figures 5D, E**). In contrast, SLC22A12 expression levels were notably elevated in ccRCC tissues compared to their corresponding adjacent normal tissues (**Figures 5A–C**). Our own RNA-Seq cohort also suggested a SLC22A12 downregulation in tumoral tissues(**Figure 5F**). Furthermore, IHC results from cancer/para-cancer pairs (**Figures 5G** and **S2**) suggest that SLC22A12 was primarily located in the plasma membranes of both cancer and normal renal tubular epithelial cells; however, it was down-regulated in cancer cells. Generally, these results collectively indicate that SLC22A12 is under-expressed in kidney cancer cells.

### SLC22A12 Restricts the Proliferation, Invasion, and Migration of RCC Cells *In Vitro*

**Figure 6:**
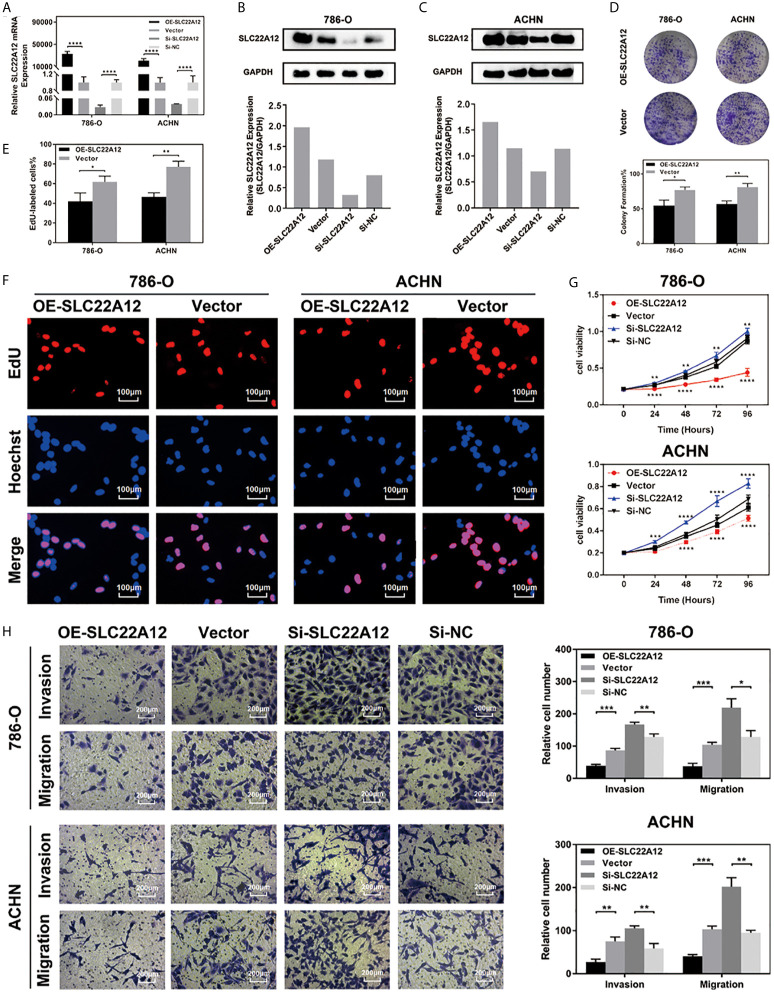
SLC22A12 promotes RCC cells proliferation, migration and invasion *in vitro*. **(A)** qRT-PCR assays and **(B, C)** immunoblotting test of SLC22A12 overexpression and knockdown in 786-O and ACHN cells. **(D)** Colony formation assays and **(E, F)** representative images (400X) of EdU assays of SLC22A12 overexpression in 786-O and ACHN cells. **(G)** CCK-8 assays examined the proliferation ability of 786-O and ACHN cells after SLC22A12 overexpression or knockdown with their corresponding negative controls. **(H)** Representative images (200X) of invasion and migration assays of 786-O and ACHN cells after SLC22A12 overexpression or knockdown with their corresponding negative controls. Data are presented as the mean ± standard deviation from three independent experiments. *p < 0.05; **p < 0.01; ***p < 0.001; ****p < 0.0001.

RCC cell lines were transfected with SLC22A12 plasmid or si-SLC22A12 to investigate the function of SLC22A12 on the pathobiology of renal cancer. The mRNA and protein expression levels increased or decreased significantly in ACHN and 786-O cells compared with the corresponding negative control (**Figures 6A–C**). Cell viability was analyzed by colony formation (**Figures 6D**), EdU (**Figures 6E, F**) and CCK-8 assays (**Figure 6G**) in both cell lines, where we observed that SLC22A12 silencing promoted cell proliferation. Moreover, transwell assays verified that the SLC22A12 expression level negatively correlates with the cells’ ability to migrate and invade (**Figures 6H**). Collectively, these results provide us with solid evidence suggesting that SLC22A12 suppresses RCC cell proliferation, migration, and invasion, which play an essential role in tumor metastasis.

### SLC22A12 Is Involved in Multiple Biological Pathways That Regulate Cellular Homeostasis and ccRCC Pathogenesis

**Figure 7:**
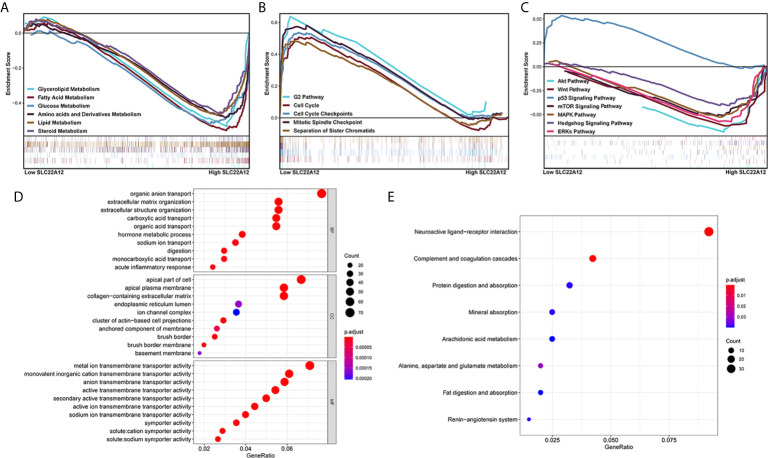
Functional enrichment analysis of SLC22A12 in ccRCC. Gene set enrichment analysis show that the activated genes are related to **(A)** multiple metabolic pathways, **(B)** cell cycle, **(C)** classic cancer-related signaling pathways. The functional differences between high-SLC22A12 and low- groups by **(D)** Gene Ontology analysis and **(E)** Kyoto Encylopedia of Genes and Genomes.

Multiple functional enrichment analyses were performed using the TCGA-KIRC cohort to study the SLC22A12 role in ccRCC pathogenesis. As demonstrated in **Figures 7A–C**, activated gene sets are associated with multiple metabolic pathways (**Figure 7A**), cell cycle (**Figure 7B**), and tumor-related signaling pathways (**Figure 7C**). The enrichment of metabolic pathways, including glycerolipid [normalized enrichment score(NES)=-2.15, p<0.001, FDR=0.053], fatty acid (NES=-2.23, p<0.001, FDR=0.047), glucose (NES=-2.02, p=0.008, FDR=0.070), amino acids and derivatives (NES=-2.20, p=0.002, FDR=0.046), and steroids (NES=-1.90, p<0.001, FDR=0.104) indicated that the role of SLC22A12 on the transport of metabolites may also partially regulate metabolic processes. The enrichment of tumor-related pathways, including Akt pathway (NES=-2.04, p=0.004, FDR=0.068), Wnt pathway (NES=-1.95, p=0.008, FDR=0.087), p53 pathway (NES=1.95, p=0.012, FDR=0.243), mTOR signaling pathway (NES=-1.74, p=0.021, FDR=0.141), MAPK (NES=-1.89, p=0.014, FDR=0.107) pathway, Hedgehog signaling pathway (NES=-1.79, p=0.042, FDR=0.129) and ERKs pathway(NES=-2.03, p=0.008, FDR=0.068), suggested that abnormally expressed SLC22A12 may activate various cancer pathways that promote ccRCC occurrence or development. The activation of PI3K and Akt pathways in SLC22A12 down-regulated cells was validated by IBT (**Figure S5**). The GO and KEGG analyses indicate that SLC22A12 is involved in the transport of multiple organic compounds, ions, and hormones, as well as extracellular structure organization (**Figures 7D, E**).

### SLC22A12 Interacts With Various Proteins That Regulate Cellular Homeostasis

To further investigate how SLC22A12 affects cellular homeostasis, we performed a PPI network analysis on the String database (https://string-db.org/) and selected the 20 proteins with the highest confidence. As shown in **Figure S3** and **Table S1**, most of the proteins are related to homeostasis maintenance. For example, SLC2A5 and SLC2A9 are involved in glucose and fructose transportation; SLC38A3 transports amino acids, while SLC5A8 and SLC16A9 transport various monocarboxylates. Also, many ion pumps essential for the exchange of substances are associated with SLC22A12. Moreover, heatmap of those genes that associated to SLC22A12 showed that most of them were expressed differentially in tumoral tissue compared to normal tissue, suggesting a completely different profile of cellular homeostasis in ccRCC (**Figure S4**). Altogether, SLC22A12 may interact with these proteins that regulate tumor cell homeostasis, thereby affecting cell proliferation, invasion, and migration.

## Discussion

RCC is among the 3% of the cancers with the highest morbidity in western countries (28). During the last two decades, RCC mortality worldwide has increased 2% per year. As a result, in 2018 within the European Union, 99,200 new cases and 39,100 kidney cancer-specific deaths were estimated (28). Regionally localized RCC has a 65–90% five-year survival rate, decreasing considerably as the tumor spread. Clear-cell RCC (ccRCC), the principal cause of renal cell carcinoma, is named after its microscopic characteristics. ccRCC cells contain a clear cytoplasm surrounded by a distinct cell membrane and round and uniform nucleus. Generally, ccRCC has a worse prognosis than papillary RCC (pRCC) and chromophobe RCC (chRCC) (29, 30), which is a big hazard for human beings. At the moment, surgery is considered the most effective treatment, although it cannot treat metastatic cancer (31–33). Targeted therapy that activates the immune system or inhibits growth factors is also employed in ccRCC patients, but drug resistance development restricts its use (34). Nevertheless, researchers have been working on new tumor-related targets involved in multiple biological processes (25, 35–42)since there is still a need for new therapeutic targets.

An ideal therapeutic target has high specificity. The idea is to attack tumor cells without affecting normal cells and target diseased organs without affecting healthy organs. Considering this, we used proteomics to study kidney-specific genes that are differentially expressed in the kidney. This means the target selected in this study is distinctive of ccRCC and has great potential as a specific drug target.

Maintenance of internal homeostasis is an essential aspect of a normal cell. Tumoral cells alter their homeostasis to adapt to their intense function, including proliferation, invasion, migration, etc. Additionally, homeostasis alterations may also support tumor development. A cellular homeostasis gene set was applied in this study that comprises various enzymes, transporters, kinases, and cytokines.

Herein, in order to find the overlapping functions of SLC22A12, we analyzed three gene sets: kidney-specific genes, cellular homeostasis genes, and survival-related DEGs. From the 19 overlapping genes, 13 were transporters, including TRPV5, SLC22A12, SLC9A4, SLC9A3, ATP6V1B1, SLC12A1, ATP6V0D2, SLC12A3, SLC4A9, ATP6V0A4, CLDN16, SLC34A1, and ATP6V1G3. It is well-known that kidney epithelial cells frequently exchange substances through diverse transporters located in the plasma membrane to preserve cellular homeostasis. The results show that kidney cell tumorigenesis is associated with cellular transporters changes.

Using univariate and multivariate Cox regression analyses, SLC22A12 came out as an effective prognostic biomarker, independent of other clinicopathological features. RCC prognosis depends on clinicopathological characteristics that include clinical symptoms, pathology, and histology; hence, transcriptomic data should also be evaluated. Our further experiments preliminarily verified the inhibitory effect of SLC22A12 expression on ccRCC.

Previous studies on SLC22A12 focused on its function as uric acid transporter. Mutations in the SLC22A12 gene are associated with diseases with abnormal serum uric acid levels, including hypouricemia (43, 44), hyperuricemia (45–47), gout (43, 45, 48), and nephrolithiasis (49). Nevertheless, SLC22A12 is not the only uric acid transporter, and it does not transport solely uric acid. Although urate processing is affected when SLC22A12 is knocked out in mice (15, 50), it was soon discovered that this effect was limited since SLC2A9 and ABCG2 played a central role in uric acid transport (50–52). Metabolomic and transcriptomic studies on SLC22A12 knockout mice revealed that URAT1 has a broader role in metabolism than previously recognized. According to Eraly et al., SLC22A12 directly interacted with urate, acetoacetate, lactate, 2-oxoglutarate, and pyruvate and affected the levels of many other essential substances, including calcium, norepinephrine, dopamine, D-fructose, glycerol, and cytidine (15). GO and KEGG analyses further proved that DEGs related to the expression of SLC22A12 were involved in the transportation of organic acid/anion, (mono)carboxylic acid, and sodium ion and hormone metabolic processes, which can affect cellular homeostasis. A similar pattern of results was obtained in the PPI network, where a strong confidence correlation was found between SLC22A12 and a large number of other transport proteins. SLC22A12 affects the organization of collagen-containing extracellular matrix. This may alter tumor growth and promote ccRCC cell proliferation, invasion, and migration, as well as activation of angiogenesis, which collectively determine the phenotype of the tumor. In summary, SLC22A12 may affect tumor progression and metastasis by affecting its cellular homeostasis.

To the best of our knowledge, the present study is the first to disclose that SLC22A12 may be a potential diagnostic and prognostic biomarker that inhibits tumor progression in ccRCC. Furthermore, this study showed that SLC22A12 up-regulation attenuates RCC cell proliferation, migration, and invasion, further proving that SLC22A12 could be used as a therapeutic target for ccRCC. However, the present study presents some limitations. First, we only verified the anti-tumor effect of SLC22A12 through *in silico* and *in vitro* experiments, without relevant *in vivo* data. Second, we have not thoroughly studied the mechanism by which SLC22A12 exerts its tumor suppressor effect. Our future research will be focused on overcoming such limitations.

In conclusion, this is the first study that demonstrates that high expression levels of SLC22A12 are associated with poor survival and low clinicopathological stage in patients with ccRCC. Furthermore, high expression levels of SLC22A12 may decrease the proliferation, migration, and invasion ability of RCC cells *in vitro*. The above results suggest that SLC22A12 is an important renal cancer biomarker and a potential highly-specific therapeutic target. SLC22A12 downregulation may impact cellular homeostasis, altering the survival of the tumor cells.

## Data Availability Statement

Publicly available datasets were analyzed in this study. This data can be found here: https://dcc.icgc.org/; https://portal.gdc.cancer.gov/; http://www.cbioportal.org/; https://xenabrowser.net/.

## Author Contributions

XZ and XY designed the study. JX and YL carried out data acquisition and analysis. JX, YL, and JL performed the majority of the experiments. JX wrote the manuscript. JX, YS, TX and QW conducted immunohistochemistry analyses. DL, HL and XZ collected the clinical samples and managed the clinical data. JL and YS contributed to bioinformatics analysis. JX, ZX and HX supplemented the experiment based on the comments made by the reviewers. XZ and XY were involved in project management. HY and XZ supervised the study. All authors contributed to the article and approved the submitted version.

## Funding

This work was supported by grants from the National Key R&D Program of China (grant no. 2017YFB1303100), the National Natural Science Foundation of China (grant nos. 81672524, 81672528 and 81874090), the Hubei Provincial Natural Science Foundation of China (grant no. 2018CFA038), the Independent Innovation Foundation of Huazhong University of Science and Technology (grant no. 118530309), the Clinical Research Physician Program of Tongji Medical College, Huazhong University of Science and Technology (grant no.5001530015), Science, Technology and Innovation Commission of Shenzhen Municipality (grant no. JCYJ20190809102415054), the Wuhan Science and Technology Plan Application Foundation Frontier Project (grant no. 2020020601012247), and the Integrated Innovation Team for Major Human Disease Program of Tongji Medical College, Huazhong University of Science and Technology.

## Conflict of Interest

The authors declare that the research was conducted in the absence of any commercial or financial relationships that could be construed as a potential conflict of interest.

## Abbreviations

RCC, renal cell cancer; TCC, transitional cell cancer; SLC22A12, Solute Carrier Family 22 Member 12; URAT1, Urate Transporter 1; RST, Renal-Specific Transporter; PPI, protein-protein interaction; DEG, differently expressed gene; TCGA, the Cancer Genome Atlas; ICGC, International Cancer Genome Consortium; RNA-seq, RNA sequencing; KIRC, kidney renal clear cell carcinoma; AJCC, American Joint Committee on Cancer; GO, Gene Ontology; qRT-PCR, reverse transcription-quantitative PCR; FBS, fetal bovine serum; IBT, immunoblotting test; RIPA, radio immunoprecipitation assay; PVDF, polyvinylidene difluoride; ECL, Electrochemiluminescence; BLAST, Basic Local Alignment Search Tool; CCK-8, Cell Counting Kit-8; GSEA, gene set enrichment analysis; KEGG, Kyoto Encylopedia of Genes and Genomes; PID, Pathway Interaction Database; FDR, false discovery rate; ROC, Receiver operator characteristic; AUC, areas under the curve; OS, overall survival; DFS, disease-free survival; KIRP, kidney renal papillary cell carcinoma; KICH, kidney chromophobesarcoma; SARC, sarcoma; NES, normalized enrichment score; ccRCC, clear-cell renal cell carcinoma; pRCC, papillary RCC; chRCC, chromophobe RCC.
