# Supplementary material for: Low Expression Levels of SLC22A12 Indicates a Poor Prognosis and Progresses Clear Cell Renal Cell Carcinoma
Source: Front Oncol. 2021 Jun 23;11:659208. doi: 10.3389/fonc.2021.659208 (PMC8262335; doi:10.3389/fonc.2021.659208)
Supplement: Supplementary file 1 [file DataSheet_1.pdf]

Supplementary files for “Low expression levels of SLC22A12 indicates a poor prognosis and progresses clear cell renal cell carcinoma”

Jiaju Xu[1†], Yuenan Liu[1†], Jingchong Liu[1], Yi Shou[1], Zhiyong Xiong[1], Hairong Xiong[2], Tianbo Xu[1], Qi Wang[1], Di Liu[1], Huageng Liang[1], Hongmei Yang[2], Xiong Yang[1*] and Xiaoping Zhang[1*]

[1]Department of Urology, Union Hospital, Tongji Medical College, Huazhong University of Science and Technology, Wuhan 430022, Hubei Province, China

[2]Department of Pathogenic Biology, School of Basic Medicine, Huazhong University of Science and Technology, Wuhan 430030, Hubei Province, China
†These authors have contributed equally to this work.

***Correspondence:**
Xiong Yang
yangxiong1368@hust.edu.cn;
Xiaoping Zhang
xzhang@hust.edu.cn

Table S1 The annotation of genes that are associated to SLC22A12 in PPI network

Gene	Annotation
ABCC4	Multidrug resistance-associated protein 4; May be an organic anion pump relevant to cellular detoxification; ATP binding cassette subfamily C
ABCG2	ATP-binding cassette sub-family G member 2; High-capacity urate exporter functioning in both renal and extrarenal urate excretion. Plays a role in porphyrin homeostasis as it is able to mediates the export of protoporhyrin IX (PPIX) both from mitochondria to cytosol and from cytosol to extracellular space, and cellular export of hemin, and heme. Xenobiotic transporter that may play an important role in the exclusion of xenobiotics from the brain. Appears to play a major role in the multidrug resistance phenotype of several cancer cell lines. Implicated in the efflux of numerous drugs.
ALPK1	Alpha-protein kinase 1; Kinase that recognizes phosphorylation sites in which the surrounding peptides have an alpha-helical conformation
CEBPD	CCAAT/enhancer-binding protein delta; Transcription activator that recognizes two different DNA motifs: the CCAAT homology common to many promoters and the enhanced core homology common to many enhancers. Important transcription factor regulating the expression of genes involved in immune and inflammatory responses. Transcriptional activator that enhances IL6 transcription alone and as heterodimer with CEBPB; Belongs to the bZIP family. C/EBP subfamily
FST	Follistatin; Binds directly to activin and functions as an activin antagonist. Specific inhibitor of the biosynthesis and secretion of pituitary follicle stimulating hormone (FSH)
LRRC16A	F-actin-uncapping protein LRRC16A; Cell membrane-cytoskeleton-associated protein that plays a role in the regulation of actin polymerization at the barbed end of actin filaments. Prevents F-actin heterodimeric capping protein (CP) activity at the leading edges of migrating cells, and hence generates uncapped barbed ends and enhances actin polymerization, however, seems unable to nucleate filaments. Plays a role in lamellipodial protrusion formations and cell migration; Belongs to the CARMIL family
NDUFA13	NADH dehydrogenase [ubiquinone] 1 alpha subcomplex subunit 13; Accessory subunit of the mitochondrial membrane respiratory chain NADH dehydrogenase (Complex I), that is believed not to be involved in catalysis. Complex I functions in the transfer of electrons from NADH to the respiratory chain. The immediate electron acceptor for the enzyme is believed to be ubiquinone. Involved in the interferon/all-trans-retinoic acid (IFN/RA) induced cell death. This apoptotic activity is inhibited by interaction with viral IRF1. Prevents the transactivation of STAT3 target genes.
PDZK1	Na^+/H^+ exchange regulatory cofactor NHE-RF3; A scaffold protein that connects plasma membrane proteins and regulatory components, regulating their surface expression in epithelial cells apical domains. May be involved

	in the coordination of a diverse range of regulatory processes for ion transport and second messenger cascades. In complex with SLC9A3R1, may cluster proteins that are functionally dependent in a mutual fashion and modulate the trafficking and the activity of the associated membrane proteins. May play a role in the cellular mechanisms associated with multidrug resistance.
SLC16A9	Monocarboxylate transporter 9; Proton-linked monocarboxylate transporter. May catalyze the transport of monocarboxylates across the plasma membrane; Solute carriers
SLC17A1	Sodium-dependent phosphate transport protein 1; Important for the resorption of phosphate by the kidney. May be involved in actively transporting phosphate into cells via Na^+ cotransport in the renal brush border membrane. Plays a role in urate transport in the kidney; Solute carriers
SLC17A3	Sodium-dependent phosphate transport protein 4; Isoform 2: voltage-driven, multispecific, organic anion transporter able to transport para-aminohippurate (PAH), estrone sulfate, estradiol-17-beta-glucuronide, bumetanide, and ochratoxin A. Isoform 2 functions as urate efflux transporter on the apical side of renal proximal tubule and is likely to act as an exit path for organic anionic drugs as well as urate in vivo. May be involved in actively transporting phosphate into cells via Na^+ cotransport; Belongs to the major facilitator superfamily. Sodium/anion cotransporter family
SLC22A12	Solute carrier family 22 member 12; Required for efficient urate re-absorption in the kidney. Regulates blood urate levels. Mediates saturable urate uptake by facilitating the exchange of urate against organic anions; Belongs to the major facilitator (TC 2.A.1) superfamily. Organic cation transporter (TC 2.A.1.19) family
SLC2A5	Solute carrier family 2, facilitated glucose transporter member 5; Functions as a fructose transporter that has only low activity with other monosaccharides. Can mediate the uptake of 2-deoxyglucose, but with low efficiency. Essential for fructose uptake in the small intestine. Plays a role in the regulation of salt uptake and blood pressure in response to dietary fructose. Required for the development of high blood pressure in response to high dietary fructose intake (By similarity); Belongs to the major facilitator superfamily. Sugar transporter (TC 2.A.1.1) family.
SLC2A9	Solute carrier family 2, facilitated glucose transporter member 9; Transport urate and fructose. May have a role in the urate reabsorption by proximal tubules. Also transports glucose at low rate; Solute carriers
SLC38A3	Sodium-coupled neutral amino acid transporter 3; Sodium-dependent amino acid/proton antiporter. Mediates electrogenic cotransport of glutamine and sodium ions in exchange for protons. Also recognizes histidine, asparagine and alanine. May mediate amino acid transport in either direction under

	physiological conditions. May play a role in nitrogen metabolism and synaptic transmission; Solute carriers
SLC5A12	Sodium-coupled monocarboxylate transporter 2; Acts as an electroneutral and low-affinity sodium (Na^+)-dependent sodium-coupled solute transporter. Catalyzes the transport across the plasma membrane of many monocarboxylates such as lactate, pyruvate, nicotinate, propionate, butyrate and beta-D- hydroxybutyrate. May be responsible for the first step of reabsorption of monocarboxylates from the lumen of the proximal tubule of the kidney and the small intestine. May play also a role in monocarboxylates transport in the retina (By similarity). Mediates electroneutral uptake of lactate.
SLC5A6	Sodium-dependent multivitamin transporter; Transports pantothenate, biotin and lipoate in the presence of sodium; Solute carriers
SLC5A8	Sodium-coupled monocarboxylate transporter 1; Acts as an electrogenic sodium (Na^+) and chloride (Cl^-)-dependent sodium-coupled solute transporter, including transport of monocarboxylates (short-chain fatty acids including L-lactate, D-lactate, pyruvate, acetate, propionate, valerate and butyrate), lactate, mocarboxylate drugs (nicotinate, benzoate, salicylate and 5-aminosalicylate) and ketone bodies (beta-D- hydroxybutyrate, acetoacetate and alpha-ketoisocaproate), with a Na^+:substrate stoichiometry of between 4:1 and 2:1. Catalyzes passive carrier mediated diffusion of iodide.
SLC9C1	Sodium/hydrogen exchanger 10; Sperm-specific sodium/hydrogen exchanger involved in intracellular pH regulation of spermatozoa. Required for sperm motility and fertility. Involved in sperm cell hyperactivation, a step needed for sperm motility which is essential late in the preparation of sperm for fertilization. Required for the expression and bicarbonate regulation of the soluble adenylyl cyclase (sAC) (By similarity); Solute carriers
UMOD	Uromodulin; Uromodulin: Functions in biogenesis and organization of the apical membrane of epithelial cells of the thick ascending limb of Henle's loop (TALH), where it promotes formation of complex filamentous gel-like structure that may play a role in the water barrier permeability (Probable). May serve as a receptor for binding and endocytosis of cytokines (IL-1, IL-2) and TNF. Facilitates neutrophil migration across renal epithelia
ZNF365	Protein ZNF365; Involved in the regulation of neurogenesis. Negatively regulates neurite outgrowth. Involved in the morphogenesis of basket cells in the somatosensory cortex during embryogenesis. Involved in the positive regulation of oligodendrocyte differentiation during postnatal growth. Involved in dendritic arborization, morphogenesis of spine density dendrite, and establishment of postsynaptic dendrite density in cortical pyramidal neurons (By similarity). Involved in homologous recombination (HR) repair pathway.

Table S2. Clinical characteristics of patients with ccRCC.

Characteristic	N (%)
Age	
Mean ± SEM, years	53±13
Sex	
Male/female	72/48
Tumor size	
Mean ± SEM, cm	5.8±3.2
Location	
Right/left	63/57
T stage	
T1a	26 (21.67)
T1b	53 (44.16)
T2a	16 (13.33)
T2b	11 (9.17)
T3	5 (4.17)
T4	2 (1.67)
Unknown	7 (5.83)
N stage	
N0	106 (88.33)
N1	14 (11.67)
M stage	
M0	109 (90.83)
M1	11 (9.17)
Fuhrman grade	
1	31 (25.83)
2	54 (45.00)
3	18 (15.00)
4	9 (7.50)
Unknown	8 (6.67)

SEM, standard error of the mean.

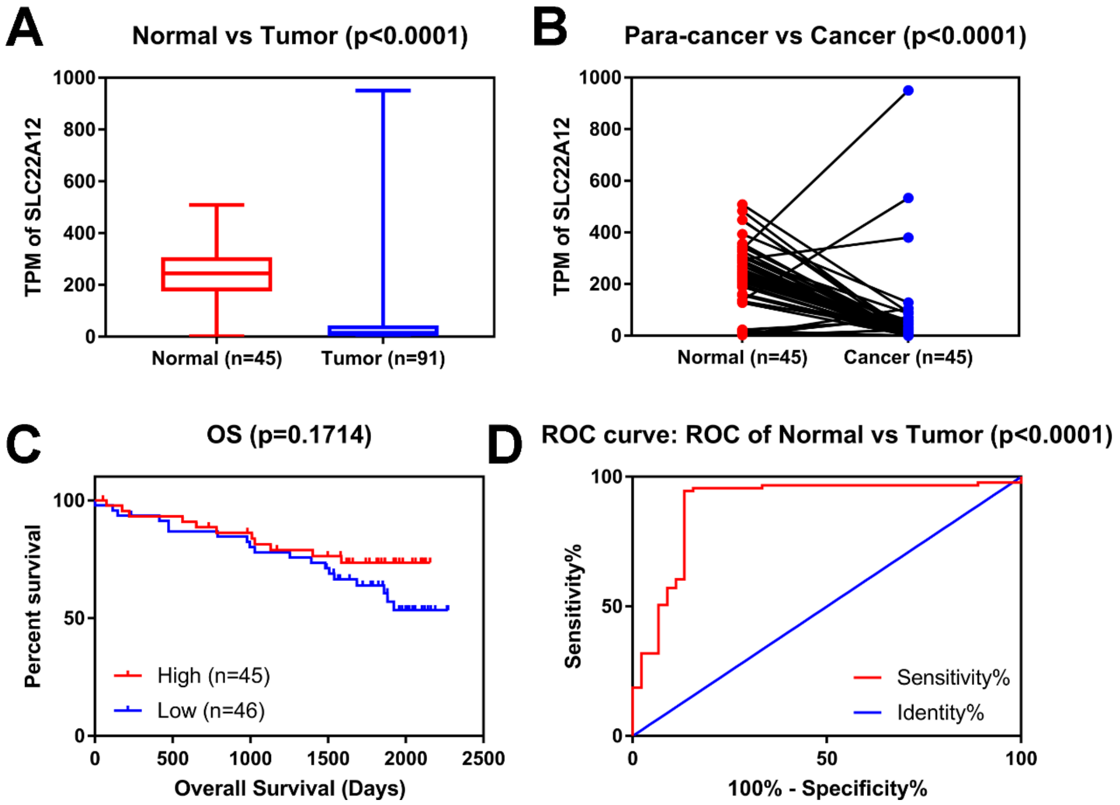

Figure S1 Transcriptome profile, survival data and ROC curve of SLC22A12 of kidney cancer in ICGC-RECA cohort. The mRNA expression levels of SLC22A12 were lower in (A) 45 RCC tissues than in 91 para-cancer tissues, (B) 45 RCC tissues than in 45 corresponding adjacent normal tissues. (C) Patients with lower SLC22A12 mRNA expression levels harbor worse OS. (D) SLC22A12 effectively discriminated between RCC and normal tissues.

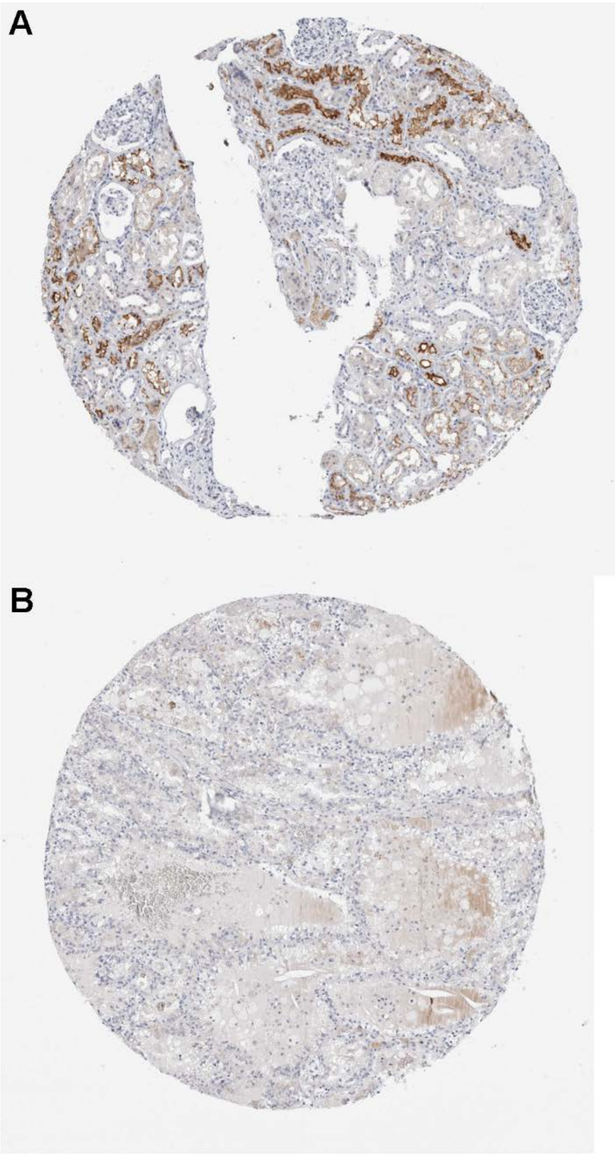

Figure S2 Relative protein expression of SLC22A12 was lower in (B) RCC tissue than para-cancer control from the same patient measured by immunohistochemical analyses. The images were downloaded from The Human Protein Atlas.

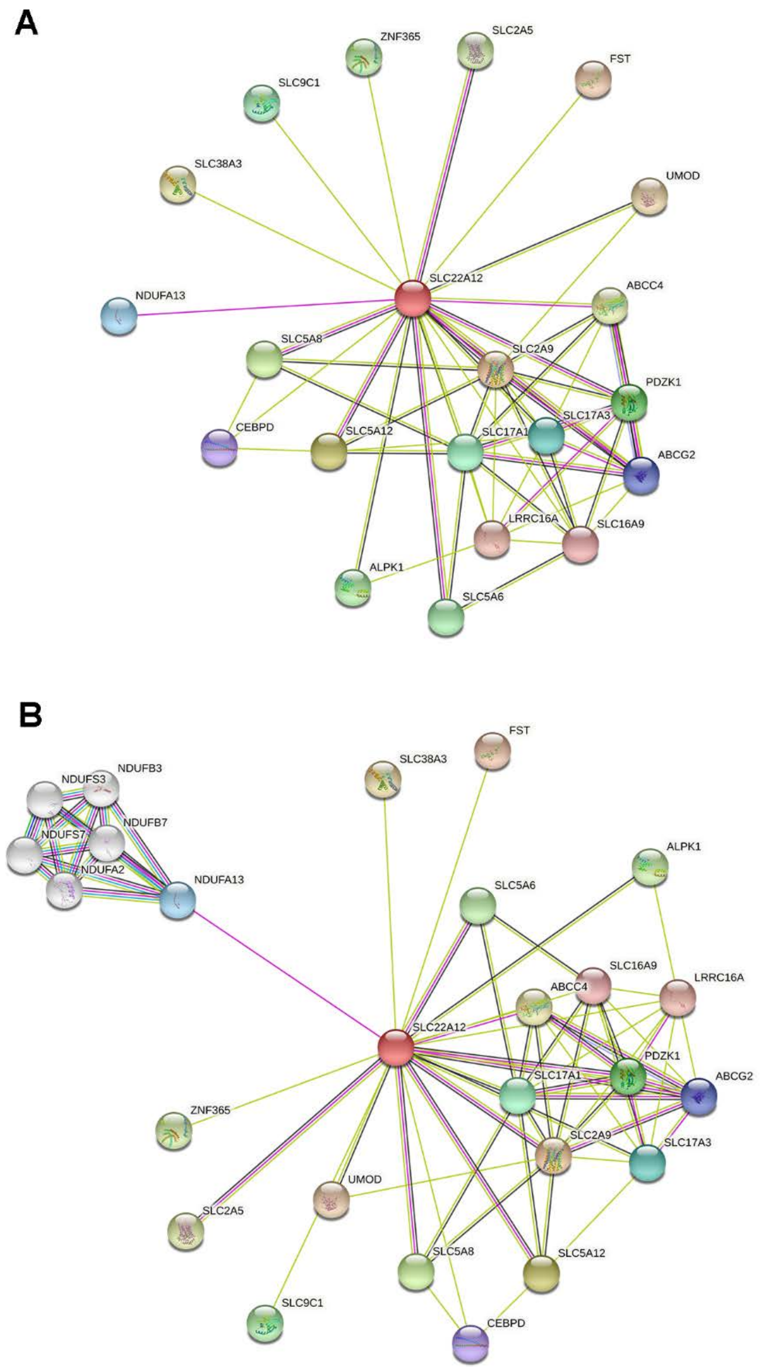

Figure S3 Protein-protein interaction network of genes that are associated to SLC22A12 with (A) single shell and (B) double shells.

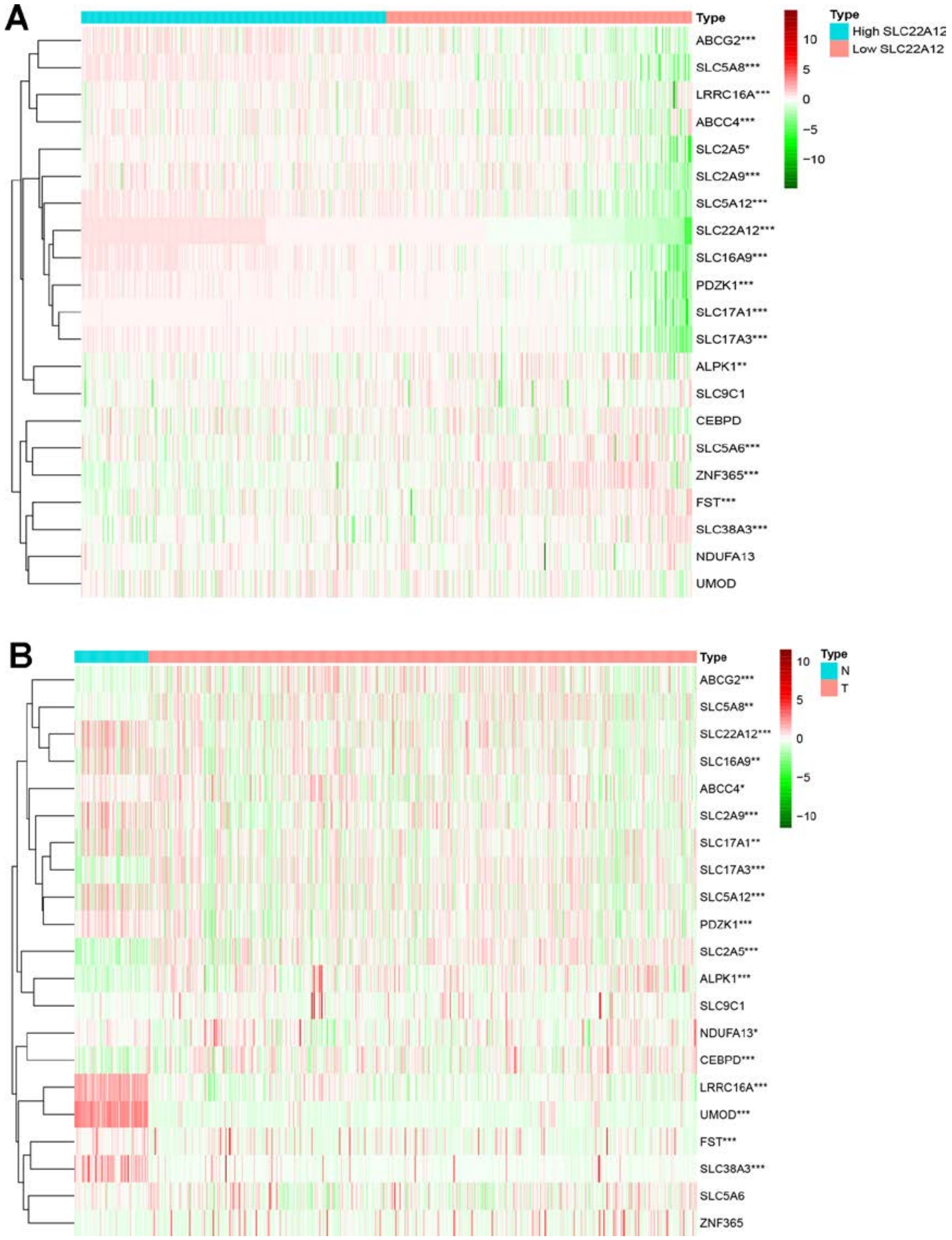

Figure S4 Heatmap of genes that are associated to SLC22A12 that are based on PPI network in TCGA-KIRC cohort. (A) High SLC22A12 vs Low SLC22A12. (B) Normal vs Tumor.

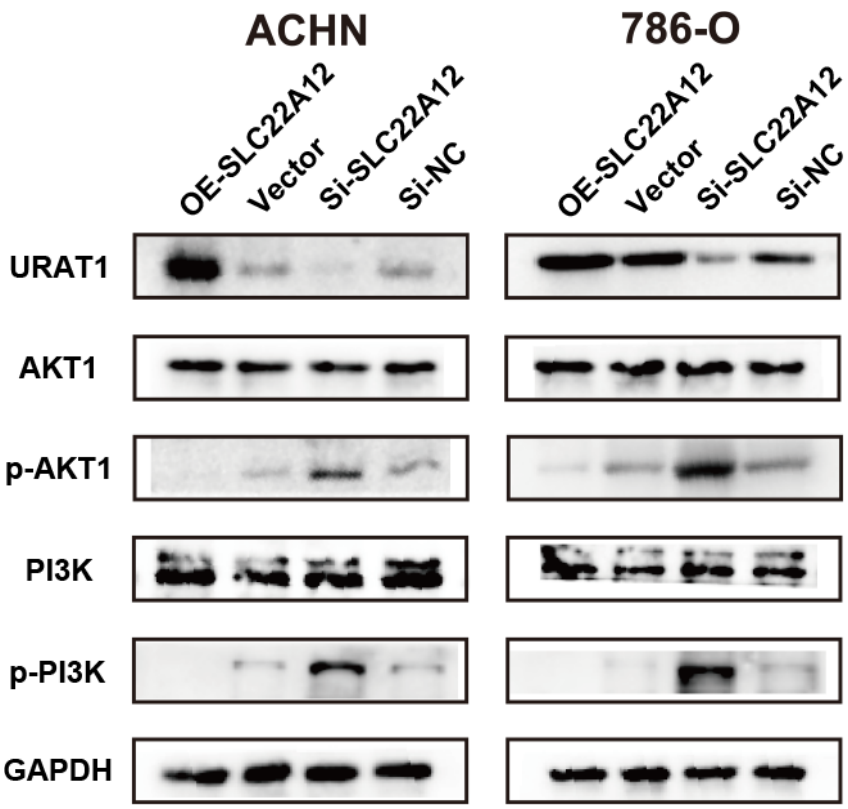

Figure S5 Overexpression of SLC22A12 inhibited PI3K/Akt pathway and vice versa.
